# Methods to Produce Nicotinic Acid with Potential Industrial Applications

**DOI:** 10.3390/ma15030765

**Published:** 2022-01-20

**Authors:** Dawid Lisicki, Kinga Nowak, Beata Orlińska

**Affiliations:** Department of Organic Chemical Technology and Petrochemistry, Silesian University of Technology, Krzywoustego 4, 44-100 Gliwice, Poland; kingnow614@student.polsl.pl (K.N.); beata.orlinska@polsl.pl (B.O.)

**Keywords:** nicotinic acid, oxidation, picoline, green chemistry, industrial synthesis

## Abstract

Nicotinic acid is a naturally occurring pyridine carboxylic acid, contained in vitamin PP, an essential nutrient for humans and animals, and used as an antipelagic agent. Nicotinic acid can be made from tryptophan by plants and animals but is usually not completely bioavailable. Industrially, nicotinic acid is produced mainly by oxidation of 5-ethyl-2-methylpyridine with nitric acid. One of the by-products of the process is nitrous oxide, a gas that is difficult to recycle and manage, with a greenhouse effect 300 times stronger than CO_2_. A new technology for the industrial production of nicotinic acid is undoubtedly necessary to meet the needs of green chemistry and not burden the environment. We carried out a literature review on ecological methods to produce nicotinic acid from commercially available raw materials such as 3-methylpyridine and 5-ethyl-2-methylpyridine, especially focusing on those methods with potential industrial applications.

## 1. Introduction

The world produced 34,000 t of nicotinic acid (NA) in 2014, 63% of which was intended as a feed additive, 22% as a food additive and for pharmaceutical use, and 15% for industrial applications [1]. On an industrial scale, 90% of NA is produced synthetically from 3-methylpyridine or 5-ethyl-2-methylpyridine. The global market size for NA was valued at USD 614 million in 2019 [2], and the largest NA producers are Lonza (18,000 t/y) and Vanetta (6000 t/y) [1].

NA is an essential nutrient for humans and helps reduce fatigue and maintain healthy skin, efficient metabolism, and mental health. The symptoms of NA deficiency are referred to as the “three D” diseases, dermatitis, diarrhea, and dementia. At the beginning of the 20th century, it was discovered that NA had a healing effect on pellagra, a disease of the skin, gastrointestinal tract, and nervous system [1]. Additionally, NA is essential for the life of animals under stress with disturbed intestinal microflora, especially farm animals, and NA deficiency leads to health problems and impairs animal reproduction and growth. For this reason, more than 60% of produced NA is used as an additive to food for poultry, pigs, fish, or domestic animals. In line with this health trend, you can find NA-enriched food products for humans, such as bread, flour, other grain products, and multivitamin drinks.

NA is available in the form of an extended-release prescription drug with the trade name Niaspan to treat hyperlipidemia and hypertriglyceridemia [3]. NA is also sold in the form of dietary supplements and mixtures for the prevention of venous insufficiency [4], leukemia [5], heart disease and anemia [6], atherosclerosis [7], insulin resistance [8], diabetic nephropathy [9], peptic ulcer disease [10], Alzheimer’s disease [11], Parkinson’s disease [12], or neoplastic diseases [13,14]. Modern preparations can be found such as cheek foils containing NA as the active substance [15]. NA has a potential anti-inflammatory and analgesic effect [16], and in dermatology, NA is used to treat acne vulgaris and rosacea [17] and is a popular ingredient in cosmetics to care for the skin and hair [18,19].

In industry, NA is used:

(1) In electroplating plants for tin plating in a sulphate bath, as well as for silvering and other electroplating applications [20,21];
(2) As an anticorrosion agent for mild steel [22];
(3) In chemical polishing of steel under high-temperature conditions;
(4) For the recovery of silver from the melting of slag [23,24];
(5) As a chemical imaging toner based on organic silver salts [25,26];
(6) As a fungicide [25,26];
(7) As an organic catalyst, including in the Hantzch reaction for the synthesis of 1,4-dihydropyridine and polyhydroquinoline derivatives [27], quinazoline [28], and 1,2,4-selenadiazoles and thiazoles under aerobic conditions [29];
(8) In the preparation of heterogenized silica-based catalysts (SBA-NA) [30] or the magnetically modified F_3_O_4_ @ NA catalyst [31];
(9) As a chelating agent in the production of VIB and VIII metal catalysts for hy-drocracking [32].

NA undergoes reactions typical for carboxylic acids and forms appropriate amides, esters, thioesters, acid halides, anhydrides, and salts. NA is also reduced to aldehyde or alcohol and hydrogenated to nipecotic acid, and it can form ionic liquids.

This paper aims to present a review on ecological methods to produce nicotinic acid from commercially available raw materials such as 3-methylpyridine and 5-ethyl-2-methylpyridine, especially focusing on those methods with potential industrial applications. First, historical methods using stoichiometric oxidizing agents as well as industrial methods of producing NA, by oxidizing 5-ethyl-2-methylpyridine with nitric acid (V), and oxidizing ammonolysis of 3-picoline, are reported. Then, new methods of obtaining NA from 3-picoline by liquid- and gas-phase oxidation are reviewed.

## 2. Historical Methods of Producing NA

**Figure 1:**
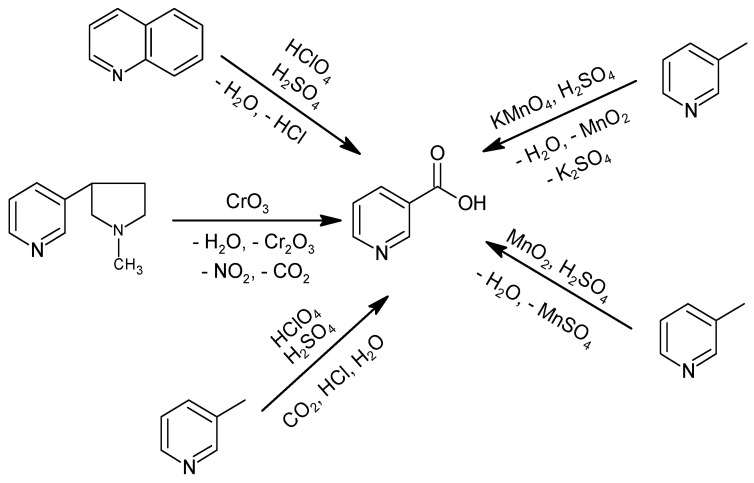
Historical methods for NA production.

Historically, NA production consisted of the oxidation of (((S)-3-[2-(*N*-methylpyrrolidine)] pyridine), quinoline, or 3-methylpyridine with stoichiometric oxidizing agents, such as KMnO_4_, MnO_2_, or HClO_4_ (Figure 1) [33,34,35,36]. Oxidation with CrO_3_ was not commercially implemented due to the high raw material cost, which requires up to 9 tons of CrO_3_ to obtain 1 ton of NA, and the carcinogenic effect of the oxidizing agent [37], which precluded use as a feed additive or in food or pharmacy [33]. Furthermore, the method using CrO_3_ produces a significant amount of inorganic waste, and the process has a low atom economy ((molecular weight of the product/total molecular weight of the substrates) × 100%) of 10%.

The process using KMnO_4_, in the presence of H_2_SO_4_ at 70–90 °C for 6 h, produced NA with 77% efficiency; however, it has only been practiced on a laboratory scale [35,38]. The improved method described in a DuPont patent used MnO_2_, also in the presence of H_2_SO_4_, but at 130 °C and a pressure of 0.1 MPa for 3–6 h, with 75% efficiency [39]. Both methods are highly wasteful, require multiple processes and unit operations to obtain NA, and have a low atom economy of 21%. Allied Dye and Chemical Corporation patented a method to obtain NA by oxidizing 3-methylpyridine or quinoline with HClO_4_ in the presence of H_2_SO_4_ and with the use of a catalyst in the form of selenium oxide or metallic selenium, preferably in the presence of bromides [34]. The oxidation of 3-methylpyridine was carried out at 250–320 °C under atmospheric pressure for 15 min, yielding NA with 50% efficiency. Quinoline oxidation was carried out at 150–190 °C for 30 min and then at 320 °C for another 30 min to decarboxylate the quinolinic acid intermediate to NA, with an efficiency of 82% [34]. These methods produce a significant amount of waste, including HCl, use a dangerous oxidizing agent, HClO_4_, and have a relatively low atom economy of 73%.

The literature also describes methods to oxidize nicotine, 3-methylpyridine, and quinoline with H_2_SO_4_ in the presence of metal catalysts [36]. By oxidizing nicotine in the range of 230–320 °C for 25–225 min, the highest efficiency for NA production was 77% and was obtained using metallic selenium, whereas the use of HgSO_4_, Bi(NO_3_)_3_, or CuSeO_3_ catalysts yielded maximum efficiencies of 46%, 34%, or 39%, respectively. The oxidation of 3-methylpyridine at 260–320 °C for 55–235 min using selenium metal as the catalyst resulted in a maximum yield of 51%, while HgSO_4_ allowed for only a 38% yield. Quinoline oxidation was carried out at 240–320 °C for 35–55 min and with a metal selenium catalyst for an almost 75% yield [36].

## 3. Industrial Methods for NA Production

**Figure 2:**
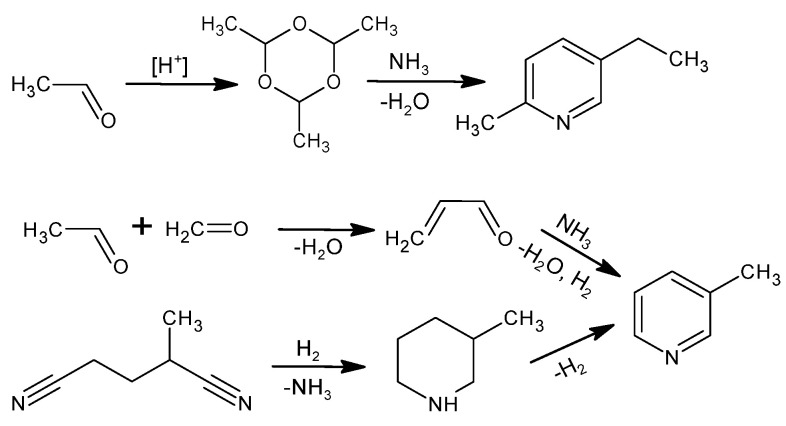
Industrial methods to produce 5-ethyl-2-methylpyridine or 3-methylpyridine.

The industrial production of NA is based on the oxidation reaction of 5-ethyl-2-methylpyridine or 3-methylpyridine. Figure 2 shows the methods for producing these raw materials [1]. When producing 5-ethyl-2-methylpyridine, an intermediate results from the trimerization of acetaldehyde to *para*-aldehyde in an acidic environment and is then reacted with NH_3_. 3-Methylpyridine is obtained from acrolein, which is reacted with NH_3_ in the gas phase; the main product is pyridine, while 3-picoline is produced as a by-product with 30–50% efficiency depending on the catalytic system [40]. The other method of obtaining 3-picoline is the catalytic hydrogenation of 2-methylglutaronitrile with a 50% yield [40]. The industrial process of obtaining 5-ethyl-2-methylpyridine is more selective and efficient compared to 3-methylpyridine, making 5-ethyl-2-methylpyridine an attractive substrate even though the atom economy (25%) for NA production is much lower using this raw material [40,41].

**Figure 3:**
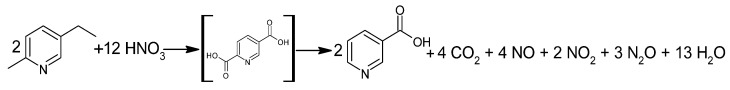
Oxidation reaction of 5-ethyl-2-methylpyridine using HNO_3_.

The Swiss Lonza group has been producing NA since 1956 [42]. One of the first technologies used by Lonza was the catalytic, liquid-phase oxidation of 5-ethyl-2-methylpyridine with HNO_3_ (Figure 3). The oxidation process was carried out at 190–270 °C at 2–8 MPa [1,43] to produce an unstable intermediate, 2,5-pyridinedicarboxylic acid (isocinchomeronic acid). The intermediate was decarboxylated at 220 °C to form NA, the crude product of which was isolated by crystallization. The total reaction time was approximately 45 min, and the first processes ran with 80% conversion and 70% efficiency [43].

The Lonza group refined their process by increasing the stoichiometric excess of HNO_3_ in the range of 25–600%. With such a large excess of acid and at temperatures below 20 °C, NA crystallized in the form of a colorless salt with HNO_3_. Once separated, the reaction mixture was dissolved in water, and the pH value was adjusted with the appropriate amount of base to release the crystalline NA.

This process forms NO that oxidizes with the air to NO_2_, which is then absorbed into water to allow HNO_3_ to return to the process [43]. Due to the highly corrosive environment, the process uses a tubular reactor made of titanium or, if operated at the upper pressure limits, a steel reactor with a titanium coating. In this way, the continuous process of NA production achieves 96% conversion and a 91% yield [43].

**Figure 4:**
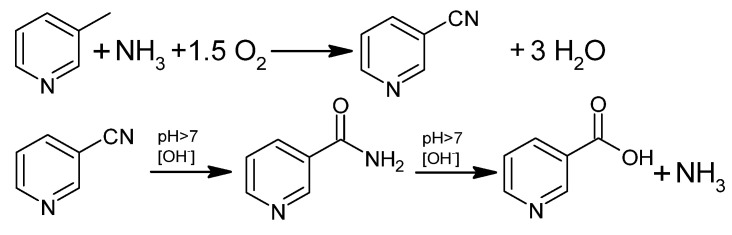
Oxidative ammonolysis of 3-picoline followed by hydrolysis to NA.

Industrially, NA is also produced by gas-phase ammoxidation of 3-picoline to 3-cyanopyridine, followed by hydrolysis to nicotinamide or NA (Figure 4). This process uses a fluidized bed reactor and a heterogeneous catalyst and has been of great interest for 30 years in Europe, Asia, and India [41,44].

In a multi-tube bed reactor, 3-methylpyridine, air, and NH_3_ react at temperatures in the range of 280–500 °C under a pressure of 0.5 MPa. Typically, the input mixture is 1–20 moles of NH_3_ and 2–20 moles of oxygen per 1 mole of 3-picoline, but the process parameters depend on the type of catalytic system. Catalytic systems most often contain vanadium(V) oxide, its mixtures, or other oxides containing metals such as titanium(IV), zirconium(VI), and molybdenum(VI) for the process carried out at 340 °C [40]. For example, ammoxidation of 3-methylpyridine in the presence of a molybdenum catalyst supported on silica gel produced 3-cyanopyridine with a 95% yield, 99% conversion, and a residence time of only 2.5 s at 380 °C [45]. Using V_2_O_5_, Lonza obtained an 83.5% yield of cyanopyridine with a conversion rate of 89.3% [40,46]. The MoO_3_-V_2_O_5_ system used by Yuki Gousei, as well as the V_2_O_5_-P_2_O_5_-SiO_2_ system used in the Koei Chemical process, led to an 82% yield of cyanopyridine with a conversion rate of 96% [40,47,48]. The V_2_O_5_-Sb_2_O_5_-TiO_2_-SiO_2_-SiC system used by Nippon Shokubai obtained an 85% yield of cyanopyridine [40]. Using the Sb_2_O_5_-V_2_O_5_-TiO_2_-montmorillonite-SiO_2_ system, Degussa obtained cyanopyridine with a yield of 90% and a raw material conversion of 94% [40,49]. The system used by Takeda Chemical is the best; V_2_O_5_-Sb_2_O_5_-Cr_2_O_3_-TiO_2_ produces an almost 99% yield of cyanopyridine at 100% conversion [40,50].

The future lies in enzymatic methods that can efficiently and selectively produce NA directly from 3-cyanopyridine [51].

Table 1 presents selected advantages and disadvantages of the oxidation of 5-ethyl-2-methylpyridine with HNO_3_.

## 4. Oxidation of 3-Methylpyridine in the Liquid Phase

**Figure 5:**
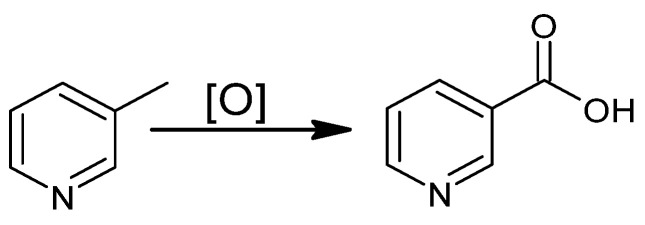
Oxidation of 3-picoline in the liquid phase.

The literature describes the oxidation of 3-methylpyridine in the liquid phase (Figure 5) with the use of environmentally friendly oxidizing agents, such as oxygen, H_2_O_2_, organic hydroperoxides, and peroxy acids, as well as non-ecological ones, such as HNO_3_.

The first attempts to oxidize 3-methylpyridine with concentrated HNO_3_ in H_2_SO_4_ medium were made by Reilly Tar & Chemical Corporation in 1945 [52]. Almost 30 years later, Lonza patented the oxidation of 3-methylpyridine to NA with HNO_3_ but without H_2_SO_4_ [53]. The reaction was carried out at 260 °C under a pressure of 5–6 MPa to obtain NA with an 89% yield and a raw material conversion rate of 69% [33,43,52]. In addition to the low technological parameters, the process is associated with the same disadvantages as discussed for the oxidation of 5-ethyl-2-methylpyridine. The process produces approximately 180 kg of N_2_O per ton of NA, and thus the method does not appear to be the best alternative, despite the lower greenhouse gas emissions and the higher atom economy of approximately 36%.

In the twentieth and twenty-first centuries, production plants in Japan, including Daicel, Mitsubishi, and Nissan, studied the oxidation of 3-picoline in the liquid phase with oxygen or air using the Amoco catalytic system, Co(II) and Mn(II) compounds with the addition of bromide salt, and most commonly with AcOH as the solvent [41].

Nissan proposed the oxidation of 3-methylpyridine with air in a catalytic system of Co(OAc)_2_, Mn(OAc)_2_, NaBr, and HCl gas in AcOH solvent [53]. The process was carried out at 80 °C and under a pressure of 10 MPa for 2 h to obtain 98% conversion and a 97% yield. The presence of the chloride derivative is key for the method and affects the yield of the process; when carried out without the presence of chlorides, the process achieves a conversion of only 60–80% depending on the conditions used [54].

Mitsubishi developed a system in which 3-methylpyridine is oxidized against 0.15–0.5% Co(OAc)_2_ and Mn(OAc)_2_ and 0.1–1.5% bromides in AcOH. The reaction was carried out at 210 °C and under 2.5 MPa pressure for 3 h to result in 93.7% conversion with 99% selectivity [55]. The company focused on developing a method where the final products could be purified from catalysts used in the reaction. However, bromides remained a problem at more than 600 ppm in the purified NA, and thus the obtained NA was passed together with water and H_2_ through a reactor with a Pd catalyst deposited on active carbon. The process was carried out at 130 °C, 0.6 MPa, for 2 h, to reduce the presence of bromides in the purified NA by more than 90% [56]. In another rendition, Daicel investigated the Co(OAc)_2_ and Mn(OAc)_2_ system with the addition of *N*-hydroxyphthalimide (NHPI) to obtain very pure NA at 150 °C and 2 MPa; unfortunately, the selectivity of the process was only 80% [56].

A variant of 3-methylpyridine oxidation that uses oxygen or air without additional metal catalysts is using a mixture of protic and aprotic solvents [57]. The best results were an 85–100% yield for NA with the 1,3-dimethyl-3,4,5,6-tetrahydro-2(1H)-pyrimidone:tetrahydrofuran (DMPU:THF) system, with oxygen serving as the oxidizing agent, where the reaction proceeded at 60–80 °C and under 1 MPa pressure for 5–10 min. A system with dimethoxyethane (DME) and THF obtained NA with only a 10% yield under analogous conditions. A solvent system with potassium *tert*-amylate (t-OAmK), hexamethylphosphoramide (HMPA) in DMPU:THF with air allowed for a maximum yield of 78% NA at 60–80 °C and 1 MPa for 5–10 min [57].

Another way to obtain NA from 3-methylpyridine is catalytic oxidation with oxygen in water under supercritical conditions. The process was carried out for 1.5 h at 380 °C and 22 MPa pressure with the use of supercritical water in the presence of MbBr_2_ as a catalyst, thereby producing NA with 30% conversion and 95% selectivity [58]. The reaction carried out with the same catalyst at 260 °C and 22 MPa allowed, in turn, the conversion of 3-picoline to NA with 83% conversion and 66% selectivity [59].

The latest research on NA production focuses on using UV-A radiation in the photocatalytic oxidation process with TiO_2_ at various pH values in an aqueous suspension. The commercially available Degussa-P25 catalyst has been used, as have the HPRT, HP60, and HP100 catalysts, which were prepared at room temperature, 60 °C, and 100 °C, respectively, with and without the presence of mineral acids. Oxidation at pH 7 for 3 h with the Degussa-P25 catalyst resulted in 88% feed conversion and 4% selectivity to NA. The HP100 catalyst with 3 M HCl catalyst produced NA with a maximum degree of conversion of 66% and with 5% selectivity. The HPRT catalyst proved to be much better with a conversion of 40% and a selectivity greater than 12%. Optimization of the pH to 2 for the Degussa-P25 catalyst obtained a maximum conversion of 14% and selectivity of 15%; in turn, the pH of 12.7 made it possible to obtain 18% selectivity, with the conversion of raw material amounting to 89%. At the same pH value of 12.7, the HP100-3M HCl catalyst produced decent results of 60% conversion and 14% selectivity [60].

There are also reports on attempts to oxidize 3-picoline in the liquid phase, in the presence of a Ag-Mn_3_O_4_ catalytic system deposited on nanorods with a diameter of 20 nm [61]. Oxidation was carried out using H_2_O_2_ with MeCN as a solvent, at 70 °C, and under atmospheric pressure for 15 h to obtain approximately 55% conversion, with selectivity to NA reaching 97%. Another example of liquid-phase oxidation is the oxidation of 3-picoline using *tert*-butyl hydroperoxide (TBHP) and oxygen as the oxidizing agents [62]. The process was performed at 80 °C for 48 h in a water solvent with reusable binaphthyl-stabilized Pt nanoparticles (Pt-BNP), which served as a catalyst to obtain 58% efficiency in producing NA.

Liquid-phase oxidation has also been investigated with peracetic acid using acetylperoxyborate (APB) as the oxidizing agent, along with the presence of Ir-Bi cluster complexes on a silica support with DME solvent [63]. The processes carried out at 65 °C for 45 min with Ir_3_Bi and Ir_5_Bi_3_ catalysts produced NA with a selectivity of 91% and 84%, respectively. Ir and Bi catalysts produced NA with 67% and 43% selectivity, respectively.

Table 2 presents selected advantages and disadvantages of the oxidation of 3-methylpyridine with air in relation to the Co/Mn/Br system.

## 5. Oxidation of 3-Methylpyridine in the Gas Phase

Gas-phase oxidation of 3-methylpyridine using heterogeneous catalysts has been known for more than 60 years, but only in the last 30 years have attempts been made to develop a commercial process. According to the principles of “green chemistry”, the process is solvent free and carried out in the presence of heterogeneous catalysts. Such late interest results from many difficulties, including easy decarboxylation and desublimation at the process temperature, the slower course of the reaction, and the formation of several by-products because of complete oxidation as compared to the liquid-phase process [33,41,51]. The most recent review on producing NA was published in 2009 [51], but since then, there have been literature reports concerning new catalyst systems and solutions for producing NA from 3-methylpyridine in the gas phase.

Researchers at the Boreskov Institute and Lonza have developed pilot processes for NA production using V_2_O_5_ and TiO_2_ oxide catalysts and proposed isolating NA by the desublimation method, a Lonza technology based on spray drying [41]. Jubilant Life Sciences used TiO_2_, V_2_O_5_, and Sb_2_O_3_ catalysts and decided to isolate NA by absorption and crystallization [64]. Additionally, researchers at the Boreskov Institute carried out a process by passing 3-methylpyridine, oxygen, and water vapor through a reactor with a catalyst deposited on a carrier at a temperature of 250–290 °C. The influence of the weight ratio of vanadium oxide to titanium oxide was also investigated, and the most advantageous ratio was 20 wt% V_2_O_5_ to 80 wt% TiO_2_, which produced NA with an 85% yield in 1.5 s, with a conversion rate of 91%. In addition to NA, the reaction produces CO_2_ and nicotinic aldehyde (PA) [65]. The reactor stream is then directed to a tubular crystallizer, where NA is separated by desublimation at 160–200 °C to generate a final product with 99.5% NA [65]. Tests on a pilot scale used the V_2_O_5_-TiO_2_ catalyst at 260–290 °C with a reaction time of less than 7 s and converted a maximum of 97% of the raw material, with a maximum yield of NA of 75% [66].

Lonza investigated catalytic systems with V_2_O_5_ deposited on a silica support or titanium oxide [33]. The raw material was almost quantitatively converted into NA, and insignificant amounts of PA were produced. Instead, the focus was on product isolation problems. After passing through the NA vapor reactor, hot air and water vapor partially condensed during absorption and in the distillation column. The solubility of NA in water is low even at 100 °C (9.8 g/100 mL) [1], meaning a considerable amount of water is needed to dissolve the obtained NA product, water which then must be removed in the later stages of the processing at much energy expense. An additional problem is the presence of ammonium ions, which reduce the amount of NA that can be crystallized due to the good solubility of ammonium nicotinate. Ammonium nicotinate decomposes above 160 °C, a fact that prompted the Lonza group to absorb the NA product on an absorption column and convert most of the NA product to an ammonium salt by adding NH_3_, and then spray drying with a drying gas (air, nitrogen, argon) at 160–250 °C [67]. The final NA product does not require additional purification and does not tend to cake.

Alternatively, Jubilant Life Sciences decided to absorb post-reaction gases in water, filter, crystallize, and dry. The process of passing 3-methylpyridine, oxygen, and water vapor through a reactor with a catalyst, consisting of TiO_2_, V_2_O_5_, and Sb_2_O_3_ deposited on a carrier, at a temperature of 250–290 °C obtained NA with almost 95% conversion and 91% selectivity [64].

A literature review of the catalysts for oxidation over the last 50 years shows progress in the design of catalytic systems that allow the reaction temperature to be decreased, as well as a marked increase in the degree of conversion of raw material and selectivity to the desired product [68]. The oldest catalytic systems were described in 1969 and consisted of SnVO_4_ and TiVO_4_ in the presence of water vapor to oxidize 3-methylpyridine with air, which produced PA and NA with a total selectivity of 75–77%. For SnVO_4_, the highest NA yield was 35%, and for TiVO_4_, it was 50%. Lower NA yields were obtained with the V-Al and V-Sn catalysts, whereas with the V-Ti catalysts developed in 1976, efficiency exceeded 50% [69]. A breakthrough in 3-methylpyridine oxidation came with the use of V-Ti systems, resulting in an 85% yield followed by a surprising 97% yield with a selectivity of more than 99%. Using promoters of molybdenum, tellurium, tin, cesium, and zirconium increased the activity of V-Ti catalysts but had little effect on selectivity. Catalysts with a lower V_2_O_5_ content (5–10%) and with additional oxides of chromium, iron, and tungsten achieved 90% efficiency. The three-component catalysts obtained NA with a yield of approximately 82% and selectivity in the range of 90–93%, regardless of the amount of vanadium. Comparison of catalysts with different compositions led to the conclusion that the efficiency of V-Ti catalysts decreased with increasing vanadium content in the range of 5–25% [68].

Degussa conducted research on the effect of the SO_4_^2–^ ion in the carrier. Catalysts containing 0.5% SO_4_^2-^ exhibited efficiencies ranging from 84 to 97%, and when the SO_4_^2–^ content increased to 1.5%, NA yield and selectivity decreased. This was most likely due to the acid–base properties of the reaction mixture and the acidic and basic surface sites of the catalysts. Acid centers can strongly influence the direction of conversion of aldehydes to acids or complete oxidation products, while basic centers result in better selectivity to aldehydes. When the SO_4_^2−^ ion concentration increased, excessive oxidation of both NA and PA likely occurred [68].

Researchers at the Bekturov Institute of Chemical Sciences have studied the effect of Al_2_O_3_, SnO_2_, and ZrO_2_ additives to vanadium catalysts [69,70]. Tests at 300 °C for a 1:1 ratio of V_2_O_5_:Al_2_O_3_ obtained NA with 64% efficiency and 94% selectivity, while a 2:1 ratio obtained NA with only 47% efficiency but almost 96% selectivity [69]. The institute also carried out a pilot-scale study, which showed greater activity and more favorable results using the V_2_O_5_-ZrO_2_-TiO_2_ system as opposed to V_2_O_5_-SnO_2_-TiO_2_, and NA was obtained with 75–77% efficiency and 90% selectivity [70].

Among vanadium-based catalysts, CrVO_4_ has been found to be most effective, with an overall yield of PA and NA of approximately 50%. The aluminum and phosphorus promoters strongly increase the overall yield to 69% and 83%, respectively. For the CrVP catalyst, NA was obtained with an efficiency of 78%, while selectivity reached 84% [68].

There are reports in the literature on the use of vanadyl pyrophosphate (VPP) as a catalyst for the oxidation of 3-picoline [71]. The process at 310 °C obtained NA with only 14% efficiency, but efficiency increased up to 36% with the addition of steam to the system. The presence of water also had a positive effect on the degree of conversion of the raw material, up to a maximum yield of 55% at 330 °C, with optimization of the raw material composition and reaction parameters. Additionally, an interesting solution was to carry out the process in the gas phase using a microwave reactor [72]. The process used a catalyst system with 20% V_2_O_5_ and 80% TiO_2_ that was heated with microwaves to achieve 95% selectivity to NA at a much lower temperature of 180 °C. This method also lowered energy consumption.

Table 3 presents selected advantages and disadvantages of the gas-phase oxidation of 3-methylpyridine with air.

## 6. Oxidative Ammonolysis of 3-Methylpyridine

Oxidative ammonolysis of 3-methylpyridine in the gas phase to 3-cyanopyridine, followed by hydrolysis to nicotinic acid amide or NA, is an industrial-scale process of great interest for 30 years [41].

One of the first published methods was a Reilly Tar & Chemical Corporation patent in 1960 [40,41]. The method consisted of passing 3-methylpyridine, air, and NH_3_ through a reactor with a catalyst bed at a temperature of 280–500 °C and a pressure up to 0.5 MPa [40]. The catalyst used in the method was most often V_2_O_5_, its mixtures, or other metal oxides [40]. The progress described in the literature optimized this catalytic system [41]. An oxide system consisting of V_2_O_5_, MoO_3_, ZrO_2_, and TiO_2_ achieved 95% conversion at 340 °C [1]. Further, a reaction using a molybdenum catalyst on silica gel, carried out at 380 °C with a residence time of 2.5 s, obtained 3-cyanopyridine in a 95% yield with a conversion of 99% [46]. Takeda Chemical used a catalyst consisting of V_2_O_5_, Sb_2_O_5_, Cr_2_O_3_, and TiO_2_ to achieve a yield of 98.6% and 100% conversion [40].

V-W-O systems use a mixture of (NH_4_)_6_[H_2_W_12_O_40_]·nH_2_O, VOSO_4_·nH_2_O, and oxalic acid and have allowed for 99.5% selectivity to 3-cyanopyridine [73]. The V-W-O system made it possible to obtain higher technological indices than the VOx-WO_3_ systems described in the literature and other vanadium-based systems.

The process of obtaining 3-cyanopyridine is perfectly adapted for industrial production, but the main purpose of the process is to obtain nicotinic acid amide. Further hydrolysis of the amide to NA is regarded as a side reaction that decreases selectivity. Nevertheless, methods have been described to successfully obtain NA.

NA has been obtained by alkaline hydrolysis with the use of catalytic amounts of 10% NaOH or KOH at 190 °C under a pressure of 1.5–2 MPa [1,74,75]. The obtained products were passed through a column with an ion exchange resin, which separated NA from its amide and resulted in a high purity of the products [76]. Another example described in the literature was catalytic hydrolysis using a 5% NaOH and MnO_2_ solution carried out in EtOH/H_2_O at 85–100 °C. After the process was complete, MnO_2_ was removed, and the solvent was evaporated [77].

The use of various types of bacteria has been studied for 20 years, including *Rhodococcus rhodochrous* that had high benzonitrilase activity and ensured 100% conversion of 3-cyanopyridine to NA [78]. Studies have also included the use of *Nocardia rhodochrous* bacteria in column bioreactors and *Bacillus pallidus* bacteria that produced a thermostable nitrilase to catalyze the hydrolysis of 3-cyanopyridine to NA without forming detectable nicotinamide [79,80]. An additional biotransformation, fungal nitrilases may convert 3-cyanopyridine to NA. The most promising biotransformation for NA production, however, seems to be plant amidases that can be used in stirred membrane bioreactors, a potentially continuous bioprocess that can be used in industry [81]. Still, the greatest challenge is the duration of enzyme activity that is used for the biotransformation.

The use of bipolar membrane electrodialysis (BMED) is a green process that can produce NA by oxidative ammonolysis [82]. The hydrolysis process yields the sodium salt of NA that can be converted to NA. Laboratory-scale tests have shown that for the highest achieved efficiency of 95.9%, the process consumes 4.14 kWh of energy per 1 kg NA.

In the literature, there are attempts to obtain NA directly in the oxidative ammonolysis process carried out in the liquid phase, using a catalyst of nanoclusters of Re_2_Sb, Re_2_Sb_2_, or Re_2_Bi_2_ deposited on a silica support [83]. The process used a toluene solvent, was carried out at 120–150 °C for 6 h, and achieved conversion rates of up to 70% with a maximum selectivity to NA of 6.9% and a selectivity to 3-cyanopyridine of approximately 80%.

Table 4 presents selected advantages and disadvantages of the oxidative ammonolysis process of 3-methylpyridine.

## 7. Other Methods of Obtaining NA

The oxidative ammonolysis of 5-ethyl-2-methylpyridine has been studied for 30 years. Problems in this process remain, and optimization is needed to limit the formation of 2,5-dicyanopyridine and increase the yield of 3-cyanopyridine. The developed methods introduce the raw material into a reactor together with oxygen, NH_3_, and steam, use catalysts V_2_O_5_, MoO_3_, and ZrO_2,_ and perform the reaction at a temperature of 350–400 °C. The use of the VTi_8_O_x_ catalyst at 350 °C allowed for a 75% yield of 3-cyanopyridine [84,85], which would then be hydrolyzed in the next step of the process. The low yield of 3-cyanopyridine, as well as problems with selective hydrolysis to NA, makes this process unjustified.

Electrochemical oxidation is another method that has been studied for several decades. The initial results of the experiments led to the formation of tar that corroded the electrodes. A system with a Pb-Ag anode with variable Ag content and a Pt cathode was used to oxidize 3-methylpyridine but did not obtain satisfactory results. Oxidation of 5-ethyl-2-methylpyridine gave 2,5-pyridinedicarboxylic acid and 6-methylnicotinic acid [86]. Oxidation of 3-methylpyridine using a cell with a Sn anode and Pt cathode increased the NA concentration to saturation of the electrolyte with the product [87]. Laboratory tests using Pb cells to oxidize 3-methylpyridine also achieved good selectivity and current efficiency. This process has been estimated to consume 11 MWh of electricity to produce 1 ton of NA [41]; this power consumption makes the method uneconomical at current energy prices.

## 8. Conclusions

The industrial process of NA production by oxidation of 5-ethyl-2-methylpyridine with nitric acid is not environmentally friendly due to gaseous by-products (NO_x_) and low atom economies. Manufacturers must meet the growing demand for the NA product and develop a method that is selective, economical, and in line with the principles of green chemistry. Hence, in recent years, many studies have been carried out, on both a laboratory scale and a pilot scale.

The process of oxidizing 3-methylpyridine in the liquid phase using green oxidizing agents has achieved relatively good selectivity to the desired NA product; the undoubted advantage is the low amount of waste and high atom economy (87%). However, these processes use solvents that contribute to corrosiveness and require processes to purify and separate the metallic catalysts in the solution, processes which are associated with high energy expenditure and water consumption.

The oxidative ammonolysis of 3-methylpyridine is a green process as the only by-product of the reaction is water. Unfortunately, the great disadvantage of this method is that it is intended to produce nicotinamide and has problems associated with implementations for the selective production of NA from 3-cyanopyridine.

Presently, the gas-phase oxidation of 3-picoline seems to be one of the best methods to produce NA with high selectivity as well as a high atom economy of 87%. This process offers very attractive advantages, such as the use of air as the oxidant, energy recovery from the exothermic reaction, and low wastage. Nevertheless, some authors mentioned that the obtained product may contain some impurities that limit its applications.

Based on the literature review, we believe that the oxidation of 3-methylpyridine to NA in the liquid phase can also be implemented in the industry. The process appears to be relatively simple and limited to a few unit operations. The use of air as an oxidizing agent and the high yields and conversions are the advantages of this method. However, the high corrosivity of the Co/Mn/Br catalytic system and resulting need to use an appropriate construction material or corrosion inhibitors should be taken into account [88].

## Figures and Tables

**Table 1:** Advantages and disadvantages of the process of obtaining nicotinic acid from 5-ethyl-2-methylpyridine.

Advantages	Disadvantages
commercial raw material	HNO_3_ as an oxidant, used in large excesses
high efficiency 91%	NOx by-products
high conversion 96%	highly corrosive reaction environment
well-known process	low atom economy (ca. 25%)
	oxidation time > 1 h
	high pressure 2–8 MPa
	high temperature 190–270 °C

**Table 2:** Advantages and disadvantages of the process of obtaining nicotinic acid by 3-methylpyridine oxidation with air in a liquid phase catalyzed by Co/Mn/Br.

Advantages	Disadvantages
commercial raw material	the technology needs further research
high efficiency 97%	highly corrosive reaction environment
high conversion 98%	oxidation time > 3 h
oxygen as an oxidant	high pressure 2–10 MPa
harmless by-products	use of a polar solvent
high atom economy (87%)	
temperature 80–210 °C	

**Table 3:** Advantages and disadvantages of obtaining nicotinic acid by 3-methylpyridine oxidation with air in the gas phase.

Advantages	Disadvantages
commercial raw material	the technology needs further research
high efficiency 91%	slightly corrosive reaction environment
high conversion 95%	high temperature > 250 °C
oxygen as an oxidant	the product impurities
harmless by-products	
high atom economy (87%)	
oxidation time < 10 min	
low pressure 0.1 MPa	

**Table 4:** Advantages and disadvantages of the process of obtaining nicotinic acid by the 3-methylpyridine oxidative ammonolysis process.

Advantages	Disadvantages
commercial raw material	low efficiency 85%
high conversion 96%	slightly corrosive reaction environment
well-known process	high temperature > 250 °C
HNO_3_ + O_2_ as an oxidant	process complexity
harmless by-products	low yield of 3-cyanopyridine hydrolysis to nicotinic acid
oxidation time < 10 min	low atom economy (66% for the preparation of 3-cyanopyridine)
low pressure 0.1–1 MPa	

## Data Availability

Data is contained within the article.
